# Program of the Alumni Association of the Southern Medical College

**Published:** 1898-07

**Authors:** 


### Society Reports.

---

### PROGRAM OF THE ALUMNI ASSOCIATION OF THE SOUTHERN MEDICAL COLLEGE.

JULY 20, 21, 22, 1898.

FIRST DAY—WEDNESDAY, JULY 20, 3 TO 5 P. M.

Opening with Prayer—Rev. A. R. Holderby, M.D , Chaplain.

Address of Welcome.

Response by John R. Shannon, A.M., M.D., of Cabaniss, Ga., Class of '93.

President's Address—"The Southern Medical College and Its History"—J McF. Gaston, Jr., of Atlanta, A.M., M.D., Class of '93.

"Suggestions on the Treatment of Some Functional Disturbances of the Gastro-Intestinal Tract in Children"—J. MacDiarmid, Dundee, Ga., Class of '91. Discussion to be opened by Dr. W. B. Parks, of Atlanta.

"Inevitable Abortion and Its Complications"—J. H. Green, M.D., Decatur, Ga. Discussion opened by Dr. J. MacDiarmid, of Dundee.

"Yellow Fever" (by request)—John Cooke Olmsted, M.D., Professor of Physiology, Southern Medical College.

"Therapeutic Suggestion"—Willis B. Parks, M.D., Atlanta, Ga , Class of '81.

"The Present Status of Massage in Medicine"—C. E. J. Smith, M.D., Atlanta, Ga., Class of '97.

SECOND DAY—THURSDAY, JULY 21, 3 TO 5 P. M.

"The Business Side of the Doctor"—C. A. Smith, M.D., Class of '98.

"God in Medical Science"—Rev. A. R. Holderby, M.D., Chaplain, Class of '94.

"The Surgical Aspect of Appendicitis" (by request)—J. B. S. Holmes, M.D., ex-Adjunct Professor of Obstetrics and Diseases of Women, Southern Medical College.

"Cases and Specimens of Operations for Appendicitis, Dermoid Cyst of the Bladder," etc.—W. A Crow, M.D , Professor of Obstetrics in the Southern Medical College.

"Scarlet Fever"—J. M. Welch, Truett, Ala., Class of '92.

Subject to be announced—John R. Shannon, A.M., M.D., Cabaniss, Ga., Class of '93.

THIRD DAY—FRIDAY, JULY 22, 3 TO 4:30 P. M.

12:00 M.—BARBECUE AT BOLTON.

Address—"The Absence of Feces in Suckling Cats"—B. B. Bell, M.D., Swift, Ga., Class of '88.

"The Application of Electricity in Medicine and Surgery"—

J. McF. Gaston, Jr., A.M., M.D., Atlanta, Ga., Class of '93.

"Malaria in a Whole Family"—C. Pelham Ward, M.D., Atlanta, Ga., Class of '94.

"Uric Acid as an Etiological Factor"—E. Van Goidtsnoven, M.D., Atlanta, Ga., Class of '83. Discussion to be opened by Dr. J. C. Olmsted.

"Labial Support"—J. D. Bradley, M.D., Kingston, Ga., Class of '88.

Report of Cases.

---

OFFICERS.

President—J. McF. Gaston, Jr., A.M, M.D., Class of '93, Atlanta. Ga.

Vice-President—George Brown, M.D., Class of '92, Atlanta, Ga.

Secretary and Treasurer—John R. Shannon, A.M., M.D., Class of 93, Cabaniss, Ga.

COMMITTEE ON MEMBERSHIP.

J. MacDiarmid, M.D., Class of '91, Dundee, Ga., Chairman.
C. A. Smith, M.D., Class of '98, Atlanta, Ga., Secretary.
John R. Shannon, A.M., M.D., Class of '93, Cabaniss, Ga.

COMMITTEE OF ARRANGEMENTS.

W. B. Parks, M.D., Class of '81, Atlanta, Ga., Chairman.
C. A. Smith, M.D., Class of '98, Atlanta, Ga.
Rev. A. R. Holderby, M.D., Class of '94, Atlanta, Ga.
E. Van Goidtsnoven, Class of '83, Atlanta, Ga.
C. Pelham Ward, M.D., Class of '94, Atlanta, Ga.

---

### SULPHONAL MANUFACTURE.

Those who follow our market-reports will have noticed that there has been great difficulty recently in obtaining sulphonal, and probably few are aware of the reasons for that. It is really because the manufacture of the drug is one of the greatest nuisances on the face of the earth. The odor of mercaptol, from which it is prepared, is equal to that of a million cats, and a whiff of the small cats' house at the Zoo is a perfume compared to the breath of the breeze that has passed over a sulphonal-factory. Messrs. Leo Fossen & Co., chemical manufacturers, Kirdorf, near Hamburg, had to pay a fine of 200m. for creating a nuisance in that neighborhood, on account of the mercaptol, and recently the permission to manufacture the article has been withdrawn from them.

